# Oxidative Stress, Antioxidants and Hypertension

**DOI:** 10.3390/antiox12020281

**Published:** 2023-01-27

**Authors:** Michael Amponsah-Offeh, Patrick Diaba-Nuhoho, Stephan Speier, Henning Morawietz

**Affiliations:** 1 Institute of Physiology, Faculty of Medicine Carl Gustav Carus, Technische Universität Dresden, Fetscherstr. 74, 01307 Dresden, Germany; 2 Department of Cardiovascular Research, European Center for Angioscience (ECAS), Medical Faculty Mannheim, Heidelberg University, 68167 Mannheim, Germany; 3 Division of Vascular Endothelium and Microcirculation, Department of Medicine III, University Hospital and Faculty of Medicine Carl Gustav Carus, Technische Universität Dresden, 01307 Dresden, Germany; 4 Department of Paediatric and Adolescent Medicine, Paediatric Haematology and Oncology, University Hospital Münster, 48149 Münster, Germany; 5 Paul Langerhans Institute Dresden (PLID) of the Helmholtz Zentrum München at University Clinic Carl Gustav Carus and Faculty of Medicine, Technische Universität Dresden, Fetscherstr. 74, 01307 Dresden, Germany; 6 German Center for Diabetes Research (DZD), 85764 München-Neuherberg, Germany

**Keywords:** hypertension, cardiovascular diseases, oxidative stress, reactive oxygen species, antioxidants, antihypertensive therapy

## Abstract

As a major cause of morbidity and mortality globally, hypertension remains a serious threat to global public health. Despite the availability of many antihypertensive medications, several hypertensive individuals are resistant to standard treatments, and are unable to control their blood pressure. Regulation of the renin-angiotensin-aldosterone system (RAAS) controlling blood pressure, activation of the immune system triggering inflammation and production of reactive oxygen species, leading to oxidative stress and redox-sensitive signaling, have been implicated in the pathogenesis of hypertension. Thus, besides standard antihypertensive medications, which lower arterial pressure, antioxidant medications were tested to improve antihypertensive treatment. We review and discuss the role of oxidative stress in the pathophysiology of hypertension and the potential use of antioxidants in the management of hypertension and its associated organ damage.

## 1. Introduction

Hypertension is a chronic medical condition in which the blood pressure is elevated, which is a major cardiovascular risk factor. Cardiovascular diseases (CVDs) are associated with thirty-one percent of global mortality [1]. Known as a silent and an invisible killer, hypertension affects at least 1.4 billion people globally [1,2]. In 2015, 1 in 4 men and 1 in 5 women had hypertension. The majority of people with hypertension are unaware of it, and fewer than 1 in 5 hypertensive individuals have sufficient therapy to control their blood pressure [1]. Hypertension resulted in an estimated global death of 10.4 million people in 2017 [3]. It is a key risk factor for coronary heart disease, stroke and chronic kidney disease, resulting in premature mortality and morbidity [4]. Therefore, a global target of 25% relative reduction in the prevalence of hypertension by 2025 has been set by the W.H.O. [5].

The dysfunction of systems that modulate cardiac, vascular and/or renal physiology contribute to the development of hypertension [6]. Hypertension is associated with multiple organ damage, and causes impairment of cardiac, vascular, kidney and brain function. Thus, the assessment of hypertension-mediated organ damage (HMOD) in these organs is crucial for the risk of clinical complications in hypertensive patients [7]. Undetected in the early stages, the presence of HMOD is common, and several manifestations of HMOD can develop in a single hypertensive individual, further enhancing the risk of severe complications [7]. Although the central nervous system plays a fundamental role in the acute regulation of arterial blood pressure via sympathetic activation, regulation of vascular tone and peripheral resistance, the renin-angiotensin-aldosterone system (RAAS) is essential in the long-term regulation of arterial pressure. However, chronic activation of RAAS largely contributes to the development and progression of hypertension, due to the sustained production of angiotensin II. This also has important therapeutic implications. Angiotensin-converting enzyme (ACE) inhibitors and angiotensin II receptor blockers (ARBs) are considered as gold standards in anti-hypertensive therapy worldwide [8,9]. Nevertheless, nearly 40% of hypertensive individuals are resistant to these standard therapies [6,10,11,12]. This indicates the critical role of other pathways such as inflammation and oxidative stress in the development and progression of hypertension, and underscores the innovative opportunity in the development of selective antioxidants as potential anti-hypertensive therapies.

In this review, we are focusing on the mechanisms of hypertension-mediated organ damage and the role of oxidative stress in the pathophysiology of hypertension. The therapeutic efficacy of antioxidants in the management of hypertension in animal and human studies is presented, and open questions are discussed.

## 2. Role of Oxidative Stress in Hypertension

**Figure 1:**
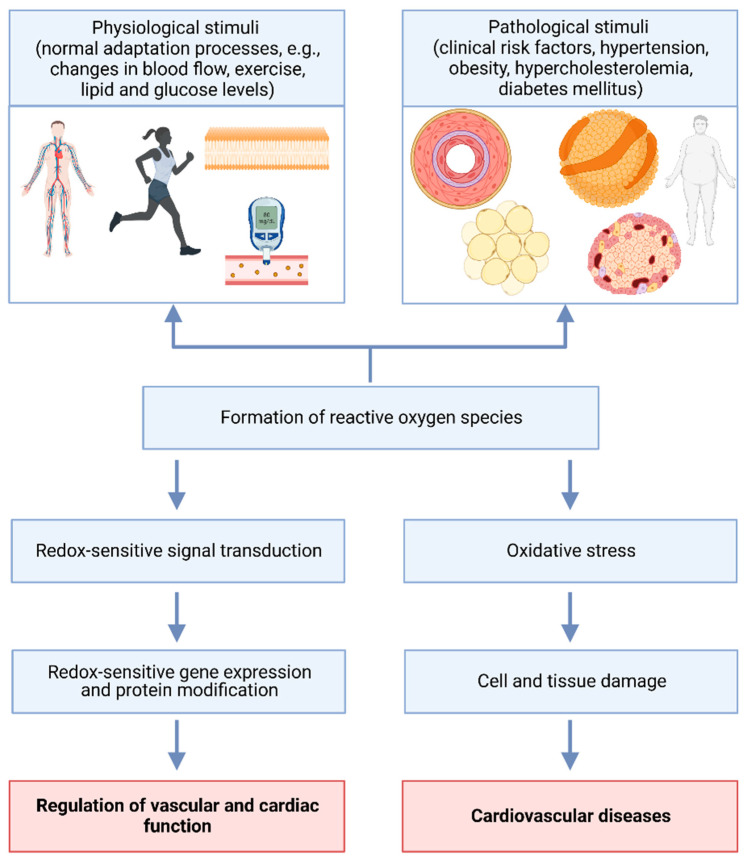
Impact of physiological and pathophysiological stimuli on the regulation of vascular and cardiac function and cardiovascular diseases. Physiological stimuli like exercise can induce redox-sensitive signal transduction and the regulation of vascular and cardiac function. Redox-sensitive signal transduction, gene and protein expression and the regulation of vascular and cardiac function are also present in patients with cardiovascular diseases, despite being altered. Clinical risk factors and the metabolic syndrome can increase levels of vasoactive substances like angiotensin II, oxidative stress, cell and tissue damage, as well as in long-term cardiovascular diseases. Created with BioRender.com.

**Figure 2:**
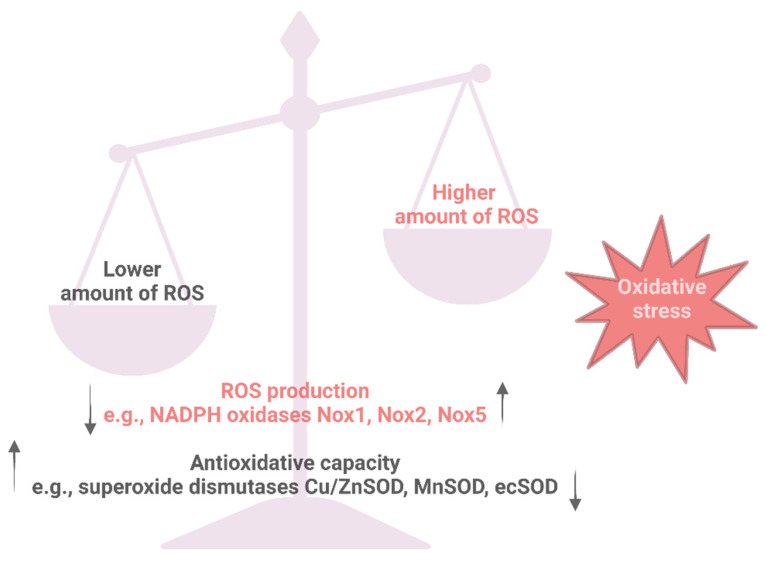
Physiological redox balance and oxidative stress. Under physiological conditions, the production of reactive oxygen species (ROS) and the antioxidative capacity are in a balance. An increased production of ROS, e.g., by NADPH oxidase (NOX) isoforms 1, 2 and 5, which is not compensated by antioxidative defense mechanisms like the different superoxide dismutase (SOD) isoforms, can lead to oxidative stress. Created with BioRender.com.

Reactive oxygen species (ROS) play an important role in the regulation of vascular and cardiac function, and the development of cardiovascular diseases (Figure 1). Pathophysiologic processes in hypertension, leading to inflammation, fibrosis and end-organ damage, are linked to the risk factor oxidative stress [13,14]. Besides pathological conditions (such as hypertension), certain genetic variations in genes related to antioxidant function may increase the risk of ROS production and oxidative stress [15,16]. Additionally, ROS production can be influenced by multiple external factors such as certain medications (chemotherapy and non-steroidal anti-inflammatory drugs), nutrition (high-fat diet, black coffee), environmental stressors (pollution and UV radiation), harmful alcohol consumption and smoking [17,18]. Oxidative stress is characterized by an imbalance between oxidants and antioxidants (Figure 2), leading to impaired redox signaling and the oxidative modification of target molecules [19,20].

The oxidative stress theory of disease is based on the idea that ROS involving (non)radicals reacts with cellular macromolecules such as DNA, RNA, proteins and lipids, causing cellular damage and cell death [21]. Interestingly, ROS have important physiologic functions. Low concentrations of ROS are important for redox regulation in maintaining endothelium integrity and vascular function [13,22,23]. The flowing blood can induce mechanical forces such as shear stress (e.g., laminar or oscillatory flow), acting on endothelial cells and affecting the formation and release of nitric oxide (NO) and ROS and the activation of signal transduction, as well as gene and protein expression that play important roles in vascular homeostasis [24,25]. Laminar flow increases endothelial nitric oxide synthase (eNOS) expression, activity and NO production, while during hypertension, oscillatory flow increases ROS formation and subsequent oxidative damage [26,27]. Exposure of endothelial cells to cigarette smoke extract can prevent the activation of the AKT/eNOS pathway, increased eNOS expression, phosphorylation and NO release in response to high laminar flow [28,29]. The type and degree of shear stress can differentially regulate the ROS- and NO-dependent signaling and remodeling of the vasculature [24,30].

Nitric oxide (NO) plays an important role in several physiological and pathophysiological processes. NO is produced by three major nitric oxide synthase (NOS) isoforms in the body. Neuronal NOS (nNOS, NOS1) is mainly found in neurons and the cardiovascular system, and is involved in the regulation of memory, learning and synaptic plasticity [16]. Inducible NOS (iNOS, NOS2) can be activated by various inflammatory stimuli and produce NO, which can mediate protective mechanisms against pathogens. Endothelial NOS (eNOS, NOS3) is mainly expressed in the cardiovascular system, and is responsible for the production of NO as the most important vasodilator [26,27]. NO can interact with superoxide anions, shifting the oxidant/antioxidant balance in favor of oxidants, increasing the formation of peroxynitrite and reducing NO bioavailability [31].

Oxidative stress has been implicated in the development of hypertension [32]. One initial hallmark is endothelial dysfunction with an impaired NO/ROS balance, supporting increased vasoconstriction, oxidation, inflammation, thrombosis and proliferation in the vessel wall [33]. The detailed molecular mechanisms of endothelial dysfunction during hypertension are still not fully resolved, even while our understanding of the role of endothelial cells in hypertension has been substantially improved in the last years [34,35]. One study found that antihypertensive therapy did not improve endothelial function in patients with essential hypertension [36], while in another study, treatment of hypertension with ACE inhibitors normalized blood pressure and restored vascular reactivity of patients [37]. In patients with essential hypertension, vitamin C improves endothelium-dependent vasodilation by restoring nitric oxide activity [38]. This supports the hypothesis that nitric oxide inactivation by reactive oxygen species contributes to endothelial dysfunction in essential hypertension [39,40]. A role of ROS in the pathogenesis of hypertension is further supported by both clinical and experimental studies [33,41,42,43,44].

**Figure 3:**
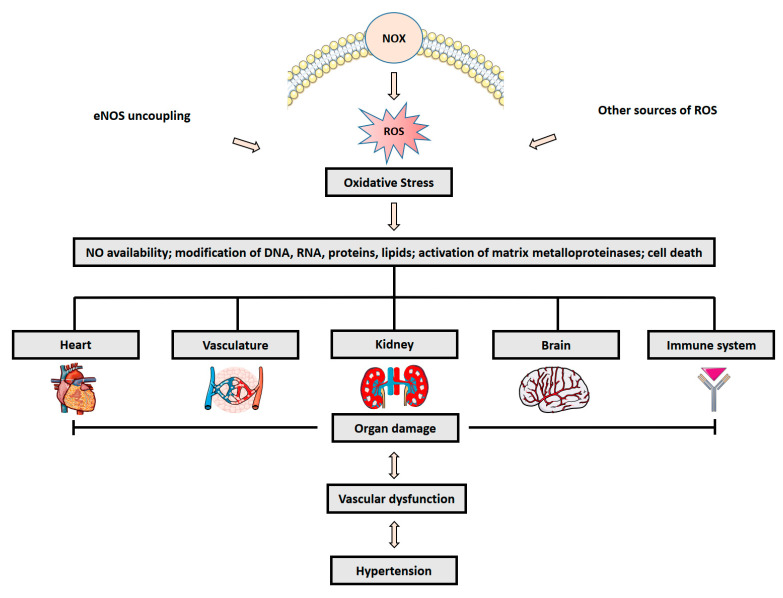
ROS formation and hypertension. Activation of NADPH oxidases (NOX) and other sources of reactive oxygen species (ROS) promotes oxidative stress, and leads to organ damage, vascular dysfunction and hypertension. Parts of figure are adapted from SMART—Servier Medical Art, Servier: https://smart.servier.com. https://creativecommons.org/licenses/by/3.0/ (accessed on 22 September 2022).

The ROS generation in the cardiovascular system is mainly mediated by nicotinamide adenine dinucleotide phosphate [NADPH] oxidases (NOX) [45,46] and other sources such as uncoupled eNOS [47], mitochondria [48] or xanthine oxidase [49] (Figure 3).

Seven isoforms of the NOX family exist: NOX1, NOX2, NOX3, NOX4, NOX5, Duox1 and Doux2 [50]. Specific NOX isoforms have important functions in the cardiovascular system. They are expressed in vasculature, kidney, brain and heart [30]. Under pathophysiological conditions NOX1, 2 and 5 are upregulated and linked to oxidative stress in hypertension [50,51]. The NOX isoforms have 6 transmembrane α-helices, a heme region and a flavoprotein homology domain on the intracellular C terminal region that contains binding sites for flavin adenine dinucleotide (FAD) and NADPH [52]. Each NOX isoform has a catalytic core unit and a transmembrane domain. The specific NOX complexes can in addition contain the subunits p22phox, p47phox/NOXA1, p67phox/NOXO1 and p40phox. The cytosolic subunits p47phox/NOXA1, p67phox/NOXO1 can be phosphorylated and translocated to the cell membrane, forming an active NOX1 or NOX2 complex. NOX5 is independent of other NOX subunits, and can be activated by calcium binding [53,54]. The generation of superoxide anions, peroxynitrite, hydroxyl radicals, nitric oxide, hydrogen peroxide and hypochlorous acid can be regulated by the cell metabolism and scavenged by antioxidants. Increasing evidence also supports a role of NOX isoforms in the regulation of cardiac intermediary metabolism [55].

NOX4 has anti-atherosclerotic and vasoprotective properties in the endothelium and protects the vasculature against oxidative stress, angiotensin II-induced aortic inflammation, tunica media hypertrophy and endothelial dysfunction [56,57,58]. Interestingly, the vasoprotective properties of NOX4 are attributed to the NOX4-derived H_2_O_2_ that can cross the membrane and act as a signaling molecule or even endothelium-derived relaxation factor (EDRF), activating downstream effectors and inducing cardiac and vascular protection [56,58]. However, NOX isoforms have also been implicated in the development and progression of hypertension [42,59]. Mice deficient in NOX1 had preserved endothelium-dependent relaxation, blunted pressure response and reduced superoxide production. Consequently, overexpression of NOX1 led to increased oxidative stress, elevated blood pressure and hypertrophic response in Ang II-induced hypertension [60,61]. NOX2 in endothelial cells contributes to Ang II-induced endothelial dysfunction, vascular remodeling and hypertension by increasing ROS production and blood pressure in transgenic mice with endothelial-specific overexpression of NOX2 [62]. In human endothelial cells, NOX2 is induced by Ang II in a dose-dependent manner [63]. Furthermore, NOX2-stimulated the production of mitochondrial superoxide by activating reverse electron transfer in Ang II-induced hypertension [64]. Ang II induced mitochondrial dysfunction via a protein kinase C-dependent pathway by activating the endothelial cell NADPH oxidase and formation of peroxynitrite. Furthermore, mitochondrial dysfunction in response to Ang II modulates endothelial NO availability [65]. Mice with fibroblast-specific deficiency of NOX2 showed reduced vascular remodeling and hypertension in response to Ang II [66]. NOX3 expression and oxidative stress is increased in the brain of stroke-prone hypertensive rats [67]. Recently, a genome-wide associated study in Eastern Chinese Han population identified a variant of NOX3, rs6557421, to have a potential effect on individual susceptibility to pulmonary hypertension [68]. The NOX4-derived H_2_O_2_ can have dose-dependent effects [69]. NOX4 was shown to be involved in the development of hypertension in Dahl salt-sensitive (DSS) rats [70]. Salt-induced hypertension in DSS rats increased H_2_O_2_ release by NOX4, and contributed to renal injury by regulating the upstream target of mammalian target of rapamycin complex 1 (mTORC1), increasing immune cell infiltration and proliferation and subsequent renal oxidative stress [71]. In humans, NOX5 might also be involved in vascular redox signaling and remodeling during hypertension [72]. Recent studies further support a link between NOX5, oxidative stress, endothelial dysfunction and systolic hypertension. Mice expressing human Nox5 in endothelial cells developed-upon aging-severe systolic hypertension and impaired endothelium-dependent vasodilation due to uncoupled NO synthase [73].

Associated organ damage as a consequence of oxidative stress in hypertension have been investigated in the vasculature [64,74], kidney [75,76], brain [77,78] and immune system [79,80]. Thus, a balance between mediators of oxidative stress and antioxidants is essential to promote physiological organ function and enhance systemic defense mechanism.

## 3. Detection and Biomarkers of ROS

Accurate assessment and detection of ROS in biological samples is challenging, due to their high reactivity and instability. Oxidative modifications of a variety of probes allow the ROS detection in biological samples. Fluorescent protein-based probes can be used to monitor changes in the levels of cytoplasmic and mitochondrial H_2_O_2_. Experimental approaches might involve the transfection of cells with plasmids or adenoviruses, leading to the formation of chimeric proteins capable of detecting ROS [81,82]. Dihydroethidium (DHE) and mitochondrial-targeted probe mitoSOX has been used to efficiently detect cellular and mitochondrial superoxide. Addition of a triphenylphosphonium group facilitates the collection of O_2_^−^ in the mitochondria, while the reaction of mitoSOX with O_2_^−^ produces 2-hydroxy-mito-ethidium, which can be measured with high performance-liquid chromatography (HPLC) [82,83,84]. In addition, short-lived free radicals in living animals can be detected using X- and L-band electron spin resonance (ESR) spectroscopy. This allows the ex-vivo analysis of tissue or blood using ESR after the infusion of cyclic hydroxylamines or nitrone spin traps. Other direct methods for ROS detection include, but are not limited to, immunospin trapping, cyclic hydroxylamine spin and boronate-based fluorescent probes [82,85].

Besides the measurement of free-radical production, ROS-modified molecules have been identified as stable biomarkers which may precisely indicate the status of local or systemic oxidative stress. Oxidative modification of lipids, proteins, DNA and RNA have been used as important biomarkers for the assessment of the redox status of human samples [86,87]. Lipids are especially susceptible to ROS-induced oxidative damage, mainly due to the presence of unsaturated double bonds. The frequently studied end products of lipid peroxidation are 4-hydroxy-2-nonenal (HNE) and malondialdehyde (MDA) [88]. In spontaneously hypertensive rats, the severity of diastolic dysfunction was associated with elevated levels of 4-HNE, which can be measured with HPLC and immune-assays with specific anti-HNE antibodies [89]. Colorimetric or fluorimetric assays can be used to detect a pink adduct complex from the reaction of thiobarbituric acid (TBA) and thiobarbituric acid reactive substances (TBARS), which include MDA, alkadienals and alkenals [90]. The ROS-induced modification of proteins can lead to reversible or irreversible alteration of their biological function. Oxidative cleavage of protein backbones yielding in carbonylation is the most common irreversible oxidative modification of proteins [90]. Additionally, the relative stability and early formation of protein carbonyls has resulted in its frequent use as a biomarker of oxidative stress and protein damage in tissues. Measurement of carbonylated proteins with methods such as HPLC and ELISA has shown elevated levels in several cardiovascular diseases [90,91]. S-glutathionylation, S-sulfenylation and S-nitrosylation are reversible protein modifications which have been identified as key signaling pathways in cardiovascular health and diseases [92]. Current developments in mass spectrometry proteomics have allowed an accurate and specific identification and quantification of oxidized proteins in several tissues. Nucleotide oxidation, DNA strand breakage, loss of bases and adduct formation are ROS-induced DNA modification can lead to mutations and DNA damage [90].

In summary, the assessment of oxidative modification of molecules in biological samples can be a valuable tool in the clinical assessment of disease severity, and support the identification of new biomarkers and potential therapies of hypertension.

## 4. Mechanism of Antioxidants and Potential Therapeutic Strategy in Hypertension

The interaction of different ROS sources and its impact on redox signalling can significantly increase oxidative stress. In contrast, antioxidants play a key functional role in reversing the detrimental effect of ROS. This includes enzymatic and nonenzymatic antioxidants like different superoxide dismutases, catalase, glutathione peroxidase, α-lipoic acid, coenzyme Q10 and vitamins [32,44,93,94]. Deciphering the molecular complexities of antioxidant interactions will support our understanding of their role in oxidative stress-induced hypertension. In this section, we discuss selected antioxidants, their potential antihypertensive and antioxidative effects and their molecular interactions.

### 4.1. Vitamins

Vitamins can play a crucial role in the improvement of endothelial dysfunction. Vitamin C and E downregulates NADPH oxidase, a major source of ROS in the vascular wall, and upregulates eNOS, thus decreasing oxidative stress and lowing BP [95]. A study analysing the role of vitamin C and E in spontaneous hypertensive rats could show an effective modulation of vascular function through regulation of eNOS and NADPH oxidases [95]. Vitamin C acts directly as antioxidant in water-soluble environments. In lipids, the tocopheroxyl radical (formed when exogenous oxidants interact with alpha-tocopherol) can be reduced by vitamin C to generate the active form of vitamin E, alpha tocopherol, thus limiting lipid peroxidation in the cell membrane, mitochondria and endoplasmic reticulum, and preserving cell integrity [96].

### 4.2. Polyphenols

Polyphenols have hydrophobic and hydrophilic domains that enable them to interact with and diffuse through biological membranes. They bind to receptors, transcription factors and enzymes involved in intracellular signalling [97]. Such different effects of polyphenols enable them to exert biological activity via mechanisms that lead to free radical scavenging, mitochondrial protection, transcription factor regulation, membrane receptor modulation and inhibition of ROS and cellular proliferation [97,98]. In hypertensive patients, quercetin could reduce BP and improve endothelial function by inhibiting the activity of ACE and the ratio of circulating ET-1 to NO [99]. Quercetin reduced blood pressure by activating Na^+^-K^+^-2Cl^−^ cotransporter 1 (NKCC1) in renal epithelial cells, elevating cytosolic Cl^−^ concentration ([Cl^−^]_c_) and downregulating gene expression of epithelial Na^+^ channel (ENaC) [100,101]. Green tee supplementation could also regulate oxidative stress, inflammation, gene expression and serum levels of vasoactive substances and modulators of BP (as Ang II and aldosterone) [102]. Significant changes were evident in downregulating the mRNA expression of ACE and ET-1 and increasing mRNA expression of eNOS [102]. Since polyphenols become free radicals after ROS scavenging, they could lead to Kelch-like ech-associated protein-1 (keap-1) thiol group oxidation and promote translocation of Nuclear factor erythroid-2 related factor-2 (Nrf2) into the nucleus [103]. Nrf2 binding to antioxidant response elements leads to the transcription of genes that encode antioxidant proteins such as hemoxygenase-1 (HO-1), superoxide dismutase-2 and glutathione peroxidase [104]. Nrf2 activation could preserve endothelial function and prevent hypertension in Ang II-induced mice [105]. Thus, the Nrf2 pathway could represent a novel area of research to explore the pleiotropic and synergistic actions of different antioxidants in reducing oxidative damage while maintaining cardiovascular function.

### 4.3. α-Lipoic Acid

In signal transduction, α-lipoic acid induction of GSH (glutathione) via transcription factor Nrf2 leads to interaction with kinases and phosphatases [106,107]. α-lipoic acid acts as a metal chelator and free radical scavenger, reduces the oxidized forms of GSH and vitamin C and E and upregulates eNOS [106].

### 4.4. N-Acetylcysteine

N-acetylcysteine (NAC) as an antioxidant acts as a reductant of disulfide bonds, a scavenger of ROS and a precursor for glutathione biosynthesis [108]. As a precursor for glutathione, it exhibits its antioxidant action directly or indirectly by lowing oxidative stress, improving insulin resistance, altering glucose metabolism, improving NO bioavailability, modulating vasoactive molecules like Ang II and hydrogen sulfide and improving renal function [109,110]. In hypertension, maternal NAC therapy could protect offspring of spontaneously hypertensive rats through the regulation of the hydrogen sulphide-generating pathway [111,112]. NAC also improved NO-dependent, alpha-adrenergic and beta-adrenergic pathways in hypertensive rats [113,114]. In a recent review, Pedre et al. discussed a new mechanism of action involving the conversion of NAC into hydrogen sulfide and sulfane sulfur species. They argued that the steady but slow delivery of intracellular cysteine by NAC allows for a low level of hydrogen sulfide and sulfane sulfur production, leading to cytoprotection through stimulating mitochondrial bioenergetics, protecting cells against oxidative damage, modulating protein function and increasing scavenging capacity [108].

### 4.5. Coenzyme Q10

A component of the electron transport chain which accepts electrons from complexes I and II and the glyceraldehyde-3-phosphate shuttle is the Coenzyme Q10 (CoQ10) [115]. CoQ10 could reduce oxidative stress and the expression of the pro-inflammatory cytokine IL-1β, thereby increasing the scavenging activity of SOD and the anti-inflammatory cytokine IL-10 in salt-induced hypertensive rats [116]. A mitochondria-targeted CoQ10 formation given orally to hypertensive rats reduced blood pressure, increased NO bioavailability and reduced cardiac hypertrophy [117]. In older patients with hypertension, low levels of CoQ10 are prevalent [118], potentially due to the increase in ROS formation during the pathogenesis of hypertension. Interestingly, several human intervention studies with CoQ10 in hypertension have demonstrated a significant reduction in blood pressure [119,120,121].

### 4.6. Superoxide Dismutase

Superoxide dismutase acts as a defence against oxidative damage. Several SOD mimetics have been developed, and their therapeutic potential has been tested in renal and cardiovascular disease models [122,123]. SOD treatment with tempol mimetic could reduce blood pressure in experimental models of hypertension, partially via the vasodilating and antihypertensive effects of increased NO bioavailability [124,125]. Tempol could also reduce vascular remodelling and decrease superoxide anion formation in salt-loaded stroke-prone spontaneously hypertensive rats [126]. Savalia and colleagues have shown that administration of nanoformulated SOD, Poly-l-lysine (PLL_50_)-polyethylene glycerol (PEG) copper/zinc superoxide dismutase (CuZnSOD) could scavenge excessive superoxide anions and decrease blood pressure in a mouse model of Ang II-induced hypertension. In cultured cells, they showed that a non-reducible cross-linked CuZnSOD nanozyme could actively deliver CuZnSOD protein to neurons without significantly inducing toxicity [127]. These data suggest that tailored antioxidant therapy of specific targets could enhance their effectiveness. Blood pressure is markedly increased in Sirt3-knockout mice, even in response to low doses of Ang II, leading to increased oxidative stress and endothelial dysfunction [128]. SIRT3 is a key mitochondrial deacetylase, and activates cyclophilin D and the mitochondrial antioxidant SOD2 [128]. Ang II and inflammation can contribute to the decline in Sirt3 activity [129]. They can influence the antioxidant capacity of a SOD mimetic, since acetylation plays an important role in the post-translational regulation of SOD2 activity by inhibiting enzyme activity at the lysine residues (K68 and K122) of SOD2 [130].

### 4.7. Antioxidants and Hypertension

Antioxidants can reduce the formation of ROS. In many human and animal models of hypertension, antioxidant activity is markedly reduced [32,33,41,42,44,131]. Glutathione and thioredoxin are impaired in hypertension. Furthermore, mild increase of bilirubin concentration within physiological levels negatively correlates with the incidence of hypertension [41,132,133]. Although a higher intake of dietary carotenoid was associated in one study with a lower risk of hypertension [134], most clinical and experimental studies, except a few (see Table 1) did not show a clear benefit of antioxidant in hypertension, atherosclerosis and cardiovascular disease [135,136,137]. Many animal models of hypertension have shown promising effects of antioxidants; however, randomized clinical trials and population studies in hypertensive patients have shown disappointing outcomes [138,139,140,141,142]. Several factors may account for these differences. First, the trial design and type of antioxidants can affect the results. A long-term exposure to increased levels of pro-oxidant factors can cause structural defects at the mitochondrial DNA level, leading to the functional alteration of several enzymes, cellular structures and aberrations in gene expression [143]. Thus, patients with several additional cardiovascular risk factors may already have irreversible oxidative damage, and expecting to reverse this with antioxidants therapy within a few years during clinical studies may be unrealistic. In addition, a long-term unhealthy lifestyle may not be necessarily compensated by a rich antioxidant food administered. To achieve elevated steady state levels in biological membranes, supplementation with lipid-soluble antioxidants such as vitamin E may require several weeks [144]. Furthermore, the biological half-life of specific antioxidants can differ, which should also be considered in experimental studies. Natural antioxidants in food or synthetic antioxidants administered as supplements may significantly differ in their mode of uptake and action. For instance, some polyphenols tend to have very low concentration levels in the blood [145], yet they work very well in the body because they activate their own antioxidant mechanisms. Furthermore, the frequent use of antioxidants in the normal nutrition might affect the responses in control groups of clinical studies. Hence, due to the lack of evidence in antioxidant use, it is not recommended as a supplementation for hypertension treatment or prevention. Nevertheless, most dietary guidelines recommend the regular consumption of a diet with antioxidant-rich fruits and vegetables, whole grain, plant fibres, salmon, nuts and reduced salt intake as a low-sodium diet, which was shown to reduce oxidative stress and improve vascular function [146,147,148,149]. A high level of fruit consumption was associated with lower blood pressure and blood glucose levels, largely independent of these and other dietary and nondietary factors, with significant lower risks of cardiovascular diseases [150].

## 5. Open Questions

In many animal models, antioxidant treatments have proven efficacious in abrogating the development of hypertension, but human studies are mostly controversial and inconclusive, which could be attributed to several factors. These include multiple risk factors like aging, comorbidities and pharmacological treatments of patients, and the frequent use of antioxidative supplements in food preparation. Additionally, in most animal models, treatment with antioxidants starts at the onset of hypertension, which is contrary to testing the anti-hypertensive effects of these antioxidants in patients with pre-existing hypertension for several years [32,241]. Recently, the US Preventive Services Task Force concluded that the current evidence is insufficient to assess the balance of benefits, and harms of the use of single- or paired-nutrient supplements (other than beta carotene and vitamin E) for the prevention of cardiovascular disease or cancer [242]. Despite these challenges, dietary antioxidant intake and polyphenols could be beneficial to reduce and prevent hypertension [148]. Potential mechanisms might also involve redox sensitive signaling, epigenetic effects, reductive stress and more. Furthermore, oxidative stress and inflammation interacts in a vicious cycle, which exacerbates the progression of hypertension and targets organ damage. Hence, the development of more selective antioxidants will be a major challenge in the field, which would provide new tools to decrease specific ROS in the vessel wall.

## 6. Conclusions

In conclusion, many experimental and clinical studies support a causal role of ROS generation and oxidative stress in hypertension and its associated target organ damage. Physiological concentrations of ROS play an important role in the maintenance of endothelial integrity and vascular function, while elevated ROS formation leads to oxidative stress, the uncoupling of eNOS and reduced bioavailability of nitric oxide. This can cause endothelial dysfunction, which promotes the progression of hypertension. Antioxidants that are more selective could decrease specific ROS generation and subsequent inflammation, which might provide an attractive therapeutic strategy in the treatment of hypertension-associated organ damage.

## Figures and Tables

**Table 1:** Studies analyzing the impact of antioxidants on hypertension in animal and human studies.

Antioxidant	Model/Subject/Study Design	Outcome of Study
Vitamin C	Spontaneously hypertensive rats (SHR) [151,152].	Blood pressure (BP) ⬇ [151,152].
	High salt-treated SHR [153].	BP ⬇, endothelium-dependent relaxation ⬆ [153].
	Hypertensive Wistar rats [154].	BP ⬇ [154].
	Stroke-prone SHR [155].	BP ⬇ [155].
	Humans, essential hypertension [38,156,157,158].	Systolic blood pressure (SBP) ⬇, endothelial vasodilation ⬆, arterial stiffness ⬇ [38,156,157,158].
	Humans, mild hypertension [159].	SBP and diastolic BP (DBP) ⬇ [159].
	Humans, elderly patients with hypertension [160].	Small ⬇ in BP and antioxidant capacity ⬆ [160].
	Humans, systemic review and meta-analysis [136].	No consistent benefit for the prevention of CVD (hypertension) [136].
	Humans, long-term risk of hypertension [138].	No clear beneficial effect [138].
	Humans, hypertension [161,162].	SBP and mean BP ⬇, endothelial vasodilation ⬆ [161,162].
	Humans, elderly patients, ambulatory BP [163].	Modest effect on BP [163].
	Humans, systemic review and meta-analysis [164].	SBP ⬇ [164].
Vitamin E	SHR, Wistar-Kyoto (WKY) rats [165,166,167].	BP ⬇ [165,166,167].
	High salt-treated Dahl salt-sensitive (DSS) rats [168].	No effect on BP [168].
	DSS rat [169].	BP ⬇ [169].
	Stroke-prone SHR [170].	BP ⬇ [170].
	Humans, hypertension and cerebral arteriosclerosis [171].	SBP ⬇ [171].
	Humans, mild essential hypertension [172].	SBP, DBP and heart rate ⬇ [172].
	Humans, sedentary elderly patients with mild systolic hypertension [173].	SBP ⬇ [173].
Vitamin C and E	Stroke-prone SHR [155].	BP ⬇ [155].
	Fructose-induced hypertensive WKY rats [174].	BP ⬇ [174].
	DOCA-salt-induced hypertensive rats [175].	SBP ⬇ [175].
	Humans, essential hypertension [157,158].	SBP, DBP ⬇, endothelial vasodilation ⬆ and arterial stiffness ⬇ [157,158].
	Humans, essential hypertension [176].	SBP and DBP ⬇ [176].
	Humans, essential hypertension [139].	No effect on BP [139].
Polyphenols		
Resveratrol	SHR, WKY rats [177,178].	BP ⬇ [177,178].
	Rats with sucrose-induced hypertension [179].	BP ⬇ [179].
	Mice with Ang II-induced hypertension [180].	BP ⬇ [180].
	Fructose-induced hypertensive rats [181].	BP ⬇ [181].
	Hypertension induced in Wistar rats [182].	SBP and DBP ⬇ [182].
	Humans, essential hypertension [140].	No significant change in BP [140].
Quercetin	Rats with sucrose-induced hypertension [179].	BP ⬇ [179].
	DOCA-salt hypertensive rats [183].	BP ⬇ [183].
	L-NAME-induced hypertensive Wistar rats [184].	BP ⬇ [184].
	SHR [185].	BP and heart rate ⬇ [185].
	Humans, randomized controlled trial [186].	DBP and mean arterial pressure ⬇ [186].
	Humans, systemic review and meta-analysis [187].	SBP and DBP ⬇ [187].
	Humans, randomized controlled trial [188,189].	SBP ⬇ [188,189].
Apocynin	SHR, WKY rats [190].	BP ⬇ [190).
	SHR [191].	BP and heart rate ⬇ [191].
	Fructose-treated hypertensive Sprague-Dawley rats [192].	SBP ⬇ [192].
	ANG II-induced hypertension in mice [193].	SBP ⬇ [193].
	DOCA-induced hypertensive rats [194].	SBP ⬇ [194].
	Dexamethasone-induced hypertension in SD rats [195].	DBP ⬇ [195].
Green Tea	SHR [196].	BP and heart rate ⬇ [196].
	Stroke-prone SHR [197].	SBP and DBP ⬇ [197].
	Salt-induced hypertensive Wistar rats [102].	SBP and DBP ⬇ [102].
	Humans, meta-analysis [198].	SBP and DBP ⬇ [198].
	Humans, systemic review and meta-analysis [199).	SBP and DBP ⬇ [199].
	Humans, systemic review [200].	SBP and DBP ⬇ [200].
	Humans, systemic review and meta-analysis [201].	SBP and DBP ⬇ [201].
(-)-Epicatechin	DOCA-salt hypertensive rats [202,203].	SBP ⬇ [202,203].
	Borderline hypertensive rats [204].	SBP ⬇ [204].
	Fructose-induced hypertensive SD rats [205].	SBP ⬇ [205].
	L-NAME-induced hypertensive Wistar rats [206].	No significant changes in SBP and heart rate [206].
	SHR, WKY rats [207].	SBP ⬇ [207].
	Humans, meta-analysis [208,209].	SBP and DBP ⬇ [208,209].
	Humans, randomized controlled trial [210].	SBP and DBP ⬇ [210].
	Humans, randomized controlled trial [141].	No significant changes in SBP [141].
*N*-acetyl cysteine	SHR [113,114,211,212,213].	SBP, mean arterial pressure, heart rate ⬇, but not DBP [113,114,211,212,213].
	Fructose-treated hypertensive WKY rats [214].	Attenuated increase in SBP [214].
	Fructose-treated hypertensive SD rats [110].	Prevented increases in SBP and DBP [110].
	L-NAME-induced hypertensive SD rats [111].	BP ⬇ [111].
	Salt-induced hypertensive Wistar rats [215].	No effect on BP [215].
	Salt-induced hypertensive DSS rats [109].	BP ⬇ [109].
	Humans, essential hypertension [216].	24hr and daytime SBP and DBP ⬇ [216].
α-lipoic acid	Fructose-treated hypertensive WKY rats [217,218].	Prevented increase in BP [217,218].
	SHR [219,220].	BP ⬇ [219,220].
	Salt-induced hypertensive WKY rats [221].	Prevented increase in BP [221].
	Salt-induced hypertensive Wistar rats [222].	BP ⬇ [222].
	High salt-induced hypertensive mice [223,224].	BP ⬇ [223,224].
	DSS rats [225].	BP ⬇ [225].
	Glucose-induced hypertensive SD rats [226,227].	Prevented increase in BP [226,227].
	Glucocorticoid-induced hypertension in SD rats [228].	Partially ⬇ SBP [228].
Coenzyme Q10	SHR [229].	BP ⬇ in older animals [229].
	Stroke-prone SHR [230].	SBP ⬇ [230].
	Salt-induced hypertensive SD rats [116].	BP ⬇ [116].
	Humans, essential hypertension [119,120,231,232,233].	SBP and DBP ⬇ [119,120,231,232,233].
	Humans, hypertension with coronary artery disease [234].	SBP and DBP ⬇ [234].
	Humans, isolated systolic hypertension [121].	SBP ⬇ [121].
	Coenzyme Q10 therapy in humans, hypertensive with metabolic syndrome [142].	No effect on SBP and DBP [142]
Superoxide dismutases	Meta-analysis using SOD mimetic tempol in hypertensive animal models [235].	BP ⬇ [235].
	EC-SOD in MCT-induced hypertensive rats [236].	Improved right ventricular SBP [236].
	Poly-l-lysine (PLL50)-polyethylene glycol (PEG) CuZn-SOD nanozyme in mice with Ang II-induced hypertension [127].	BP ⬇ [127].
	Melon SOD in SHR [237].	BP ⬇ [237].
	Tempol in hypertension of Wistar rats [124].	SBP ⬇ [124].
	Tempol in fructose-induced hypertensive SD rats [125].	BP ⬇ [125].
	Tempol in salt-loaded stroke-prone SHR [126].	SBP ⬇ [126].
	Tempol in advanced-stage stroke-prone SHR [238,239].	No effect on SBP [238,239].
	TAT-SOD in humans, essential hypertension [240].	SBP and DBP ⬇ [240].

Abbreviations: ANG, angiotensin; BP, blood pressure; DBP, diastolic BP; DOCA, deoxycorticosterone acetate; DSS, Dahl salt-sensitive; EC-SOD, extracellular superoxide dismutase; L-NAME, L-NG-Nitro arginine methyl ester; MCT, monocrotaline; SBP, systolic BP; SD, Sprague-Dawley; SHR, spontaneous hypertensive rats; SOD, superoxide dismutase; WKY rats, Wistar-Kyoto rats. ⬇, decrease/d; ⬆, increase/d.
